# Severe Varicella-Zoster Virus Pneumonia in an Unvaccinated Patient With Rheumatoid Arthritis: A Case Report and Literature Review

**DOI:** 10.7759/cureus.74183

**Published:** 2024-11-21

**Authors:** Takahiro Mamiya, Hironori Kobayashi, Shunta Takeuchi, Mayumi Tago, Tadasuke Ikenouchi

**Affiliations:** 1 Education and Training, Handa City Hospital, Handa, JPN; 2 Respiratory Medicine, Handa City Hospital, Handa, JPN

**Keywords:** immunocompromised, janus kinase inhibitors, recombinant zoster vaccine, reinfection, varicella

## Abstract

The global prevalence of rheumatoid arthritis (RA) is increasing, resulting in an increased use of Janus kinase (JAK) inhibitors. Several cases of varicella-zoster virus (VZV) pneumonia in patients with RA have been reported. However, to our knowledge, no reports have demonstrated conclusive evidence of VZV reinfection in this patient population. This case report describes a 52-year-old female with RA who developed severe VZV pneumonia. The patient was treated with a combination of methotrexate, baricitinib, and iguratimod. She had a history of chickenpox during childhood and had not been vaccinated against VZV. Two weeks after her family member was infected by shingles, the patient developed multiple vesicles throughout her body. The patient was diagnosed with VZV reinfection based on the history and serological testing. She was admitted and treated with intravenous acyclovir for the disseminated VZV infection. Despite treatment, her condition rapidly deteriorated, progressing to acute respiratory distress syndrome. Chest computed tomography revealed diffuse bilateral ground-glass opacities, nodules, and consolidations, consistent with VZV pneumonia. The patient required high-flow nasal cannula oxygen and steroid therapy. Following the administration of acyclovir and steroids, the patient gradually improved and was discharged on the 15th day of admission. This case highlights the risk of severe VZV infection in patients with RA, particularly in those treated with JAK inhibitors. This underscores the importance of the VZV vaccination in this population. Despite the current guidelines recommending VZV vaccination, vaccination rates among immunosuppressed patients remain inadequate. Given the potential for VZV reinfection, vaccination is recommended, regardless of previous VZV infection status.

## Introduction

The worldwide increase in rheumatoid arthritis (RA) cases has led to greater utilization of Janus kinase (JAK) inhibitors [1,2]. Because immunosuppressed individuals face an elevated risk of herpes zoster infection, the Centers for Disease Control and Prevention recommends a recombinant zoster vaccine (RZV) [3]. However, RZV is not yet actively administered in Japan [4]. Varicella-zoster virus (VZV) reactivation is well-known as shingles, whereas reinfection describes a second episode of varicella caused by external exposure [5]. Although reinfection was thought to be less common, a surveillance study has found that 4.5%-13.3% of participants reported VZV reinfection [6]. Several cases of VZV pneumonia in patients undergoing immunosuppressive therapy for RA have been reported [7-10]. However, to the best of our knowledge, no previous studies have shown definitive evidence of VZV reinfection in this patient population. We report a case of severe VZV pneumonia in an unvaccinated patient with RA who was treated with immunosuppressive therapy, including methotrexate, baricitinib (a JAK inhibitor), and iguratimod. In addition, we provide a literature review.

## Case presentation

**Figure 1:**
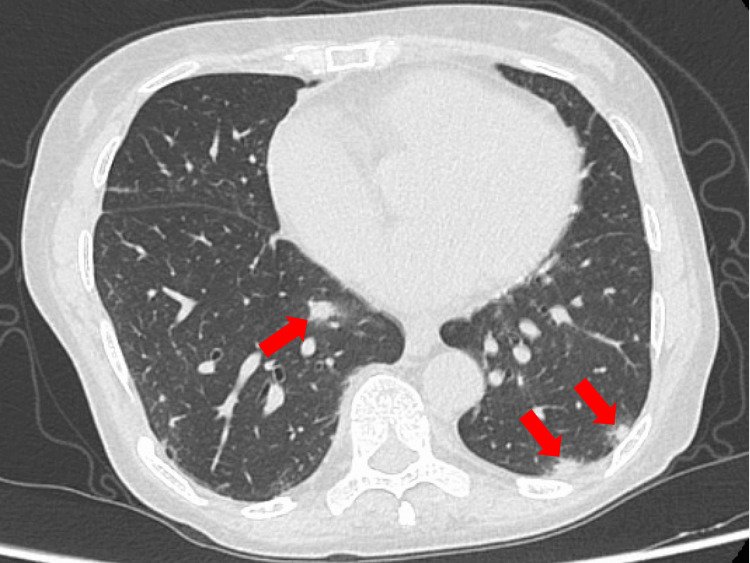
Chest computed tomography imaging on the day of admission Chest computed tomography revealed multiple micronodules in both lungs.

A 52-year-old female with a history of RA was treated with methotrexate (10 mg/week), baricitinib (4 mg/day), and iguratimod (50 mg/day). The patient had varicella as a child and had not received vaccination against VZV. She presented with multiple acute-onset vesicles throughout her body, including her head. The vesicles developed two weeks after exposure to her family member, who had been diagnosed with shingles. VZV infection was confirmed by a positive vesicular fluid antigen test. Titers of VZV-IgM and VZV-IgG were 0.29 and 4.2, respectively, consistent with previous VZV infections. Chest computed tomography (CT) revealed multiple micronodules in both lung fields (Figure 1).

**Figure 2:**
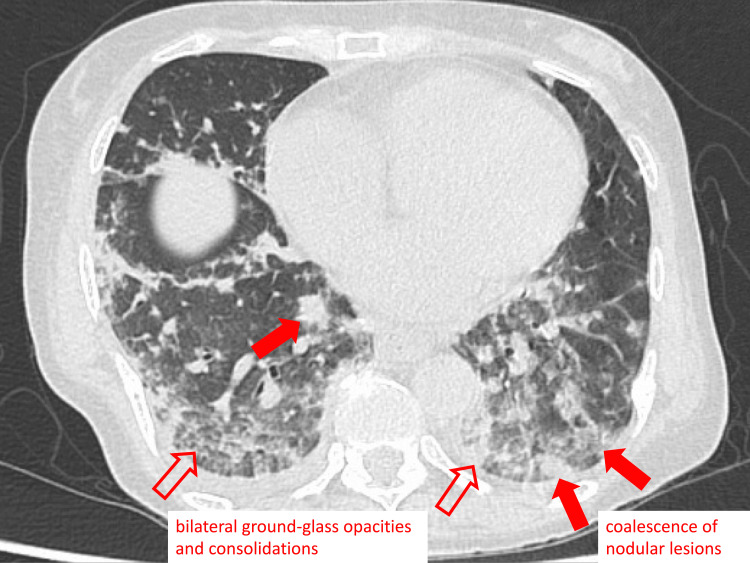
Chest computed tomography imaging on the third day of admission Chest computed tomography revealed diffuse bilateral ground-glass opacities, coalescence of nodular lesions, and consolidation.

She was admitted for the treatment of a disseminated VZV infection and received intravenous acyclovir (10 mg/kg every 8 h). As VZV can be transmitted from person to person via airborne transmission, all medical staff wore N95 respirators, and the patient was managed in a negative pressure room. On admission, her vital signs were stable, with a respiratory rate of 12 breaths/min and an oxygen saturation of 99% on room air. However, her respiratory condition rapidly deteriorated two days after admission; her respiratory rate increased to 30 breaths/min, and her oxygen saturation decreased to 85% in room air. Repeat chest CT showed diffuse bilateral ground-glass opacities, coalescence of nodular lesions, and consolidations, which were consistent with VZV pneumonia (Figure 2) [11].

**Figure 3:**
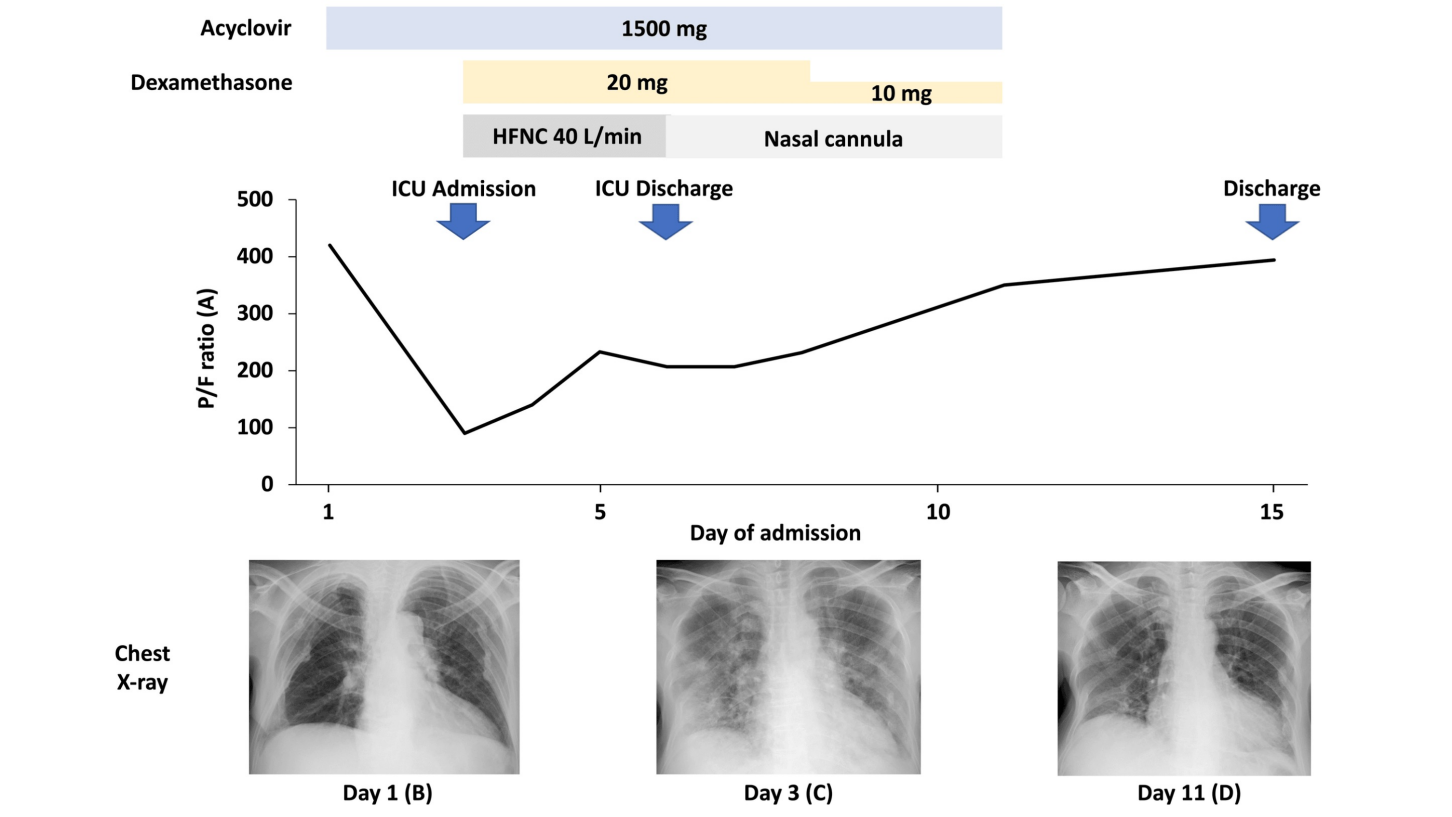
Clinical course HFNC, high-flow nasal cannula; P/F ratio, a ratio of the partial pressure of arterial oxygen to the fraction of inspired oxygen; ICU, intensive care unit. The patient was treated with acyclovir (10 days) and dexamethasone (8 days) to treat disseminated varicella-zoster virus infection and acute respiratory distress syndrome. Although the P/F ratio (A) was over 400 on admission, it dropped to 89.8 two days after admission. The P/F ratio (A) gradually improved with high-flow nasal cannula oxygen therapy and dexamethasone. The patient was weaned off oxygen on the 11th day of admission. The initial chest X-ray on admission (B) was normal. Diffuse ground glass opacities were observed on day 3 (C). Radiographic findings showed marked improvement on day 11 (D).

The patient was admitted to the intensive care unit, and high-flow nasal cannula oxygen therapy was initiated at a rate of 40 L/min. Oxygenation saturation continued to worsen over time, with the ratio of partial pressure of arterial oxygen to fraction of inspired oxygen decreasing to 89.8. High-flow nasal cannula oxygen therapy with a fraction of inspired oxygen (FiO2) of 0.8 was required to maintain an oxygen saturation of 90%. Dexamethasone was administered (20 mg/day) to treat the acute respiratory distress syndrome [12]. With continued therapy, her respiratory condition gradually improved. On the 6th day after admission, the patient was switched to a nasal cannula and was discharged from the intensive care unit. She was weaned off oxygen on the 11th day of admission and was discharged on the 15th day. ​​​The clinical course of this case is summarized in Figure 3.

## Discussion

This case report describes severe VZV pneumonia in an unvaccinated patient with RA who was receiving treatment with a JAK inhibitor. This case presents two notable aspects: the development of severe VZV pneumonia in an unvaccinated RA patient treated with a JAK inhibitor and VZV reinfection despite a history of childhood varicella.

JAK inhibitors are increasingly used in the treatment of RA, enabling clinical remission in many patients [1,2]. However, they are also associated with an increased risk of herpes zoster, particularly in the Japanese population [1,13]. Several cases of severe VZV pneumonia in patients with RA undergoing disease-modifying antirheumatic drugs have been reported [7-10]. These cases, including the present case, demonstrated rapid deterioration and life-threatening complications. RZV significantly reduces the risk of VZV infection in immunocompromised patients who are at substantial risk of the infection [14]. The combination of the increased risk of VZV infection in immunocompromised patients and the effectiveness of RZV underscores the importance of vaccination in the management of patients undergoing RA treatment.

We reviewed the literature on five reported cases of severe VZV pneumonia in patients with RA, including the present case [7-10]. The patients received various combinations of RA treatments, including methotrexate, iguratimod, JAK inhibitors, and steroids. Two lacked documentation regarding VZV vaccination, whereas the remaining three were confirmed as unvaccinated. Two patients had a history of previous VZV infection, two had no history of infection, and one was unknown. Treatment approaches were consistent in some aspects but varied in others; all patients received acyclovir, three were treated with antibiotics, and three were administered steroids. Despite these interventions, one fatality was reported. The deceased patient had a history of diffuse interstitial lung disease and developed respiratory failure complicated by *Pseudomonas aeruginosa* respiratory infection (Table 1). 

**Table 1:** Severe varicella pneumonia in patients with rheumatoid arthritis VZV: varicella-zoster virus; RA: rheumatoid arthritis; ABPC/SBT: ampicillin/sulbactam; NA: not available

First author [ref.], year	Age (years)/ sex	RA Treatment	Vaccination History	VZV History	VZV Treatment	Outcome
Mamiya (Current study)	52/ female	Methotrexate, iguratimod, baricitinib	Not vaccinated	Previous infection	Acyclovir, dexamethasone	Survival
Kobayashi [10], 2023	66/ female	Methotrexate, iguratimod, prednisolone	Not vaccinated	No history	Acyclovir, ABPC/SBT, methylprednisolone	Survival
Ito [8], 2022	72/ female	Methotrexate, iguratimod	Not vaccinated	Previous infection	Acyclovir, valacyclovir, ABPC/SBT, vancomycin	Survival
Abe [7], 2018	68/ female	Tofacitinib	NA	No history	Acyclovir	Survival
Urrutia [9], 2022	78/ male	Methotrexate, prednisone	NA	NA	Acyclovir, antibiotic treatment with antipseudomonal, corticosteroid	Dead

Despite a history of childhood varicella, VZV reinfection in the present case was noteworthy. While VZV reactivation is well documented in immunocompromised individuals, reinfection is less common. However, a surveillance study found that 4.5%-13.3% of participants reported reinfection with VZV. Of the individuals who experienced two episodes of varicella, 44.9% reported that at least one family member had experienced a recurrent varicella infection [6]. One case of VZV reinfection in a patient with RA undergoing immunosuppressive therapy has been previously reported [8]. However, this report has a significant limitation in its diagnosis of VZV reinfection. The authors made their diagnosis based only on antibody titer patterns, without any documented exposure to VZV. This is problematic because antibody titer patterns alone cannot definitively distinguish between reactivation and reinfection [15].

The present case provides strong evidence of VZV reinfection. The patient had a history of VZV infection, and the antibody levels were consistent with past exposure. The patient developed symptoms 14 days after exposure to her family member with herpes zoster, matching the typical incubation period of VZV (10-21 days). This clear timeline of exposure and symptom onset strongly suggested reinfection rather than reactivation. This suggests that patients with RA receiving immunosuppressive therapy, particularly JAK inhibitors, may be at risk of VZV reinfection even with prior exposure. This observation further supports the importance of RZV in this population, regardless of infection history. RZV is recommended for preventing VZV infections in immunocompromised patients [2,3]. However, vaccination rates among these patients remain low owing to the limited awareness of RZV [4]. This gap underscores the need for improved education and the implementation of vaccination strategies in RA management.

## Conclusions

To the best of our knowledge, this is the first documented case of VZV pneumonia in an unvaccinated patient with RA who was receiving a JAK inhibitor and had a clear history of VZV exposure, resulting in reinfection. This report underscores the potential severity of VZV infections in RA patients treated with JAK inhibitors and emphasizes the critical importance of VZV vaccination in this population. The unique aspects of this case contribute to the existing literature on VZV infections in patients with RA.
